# Synergistic Efficacy of CDK4/6 Inhibitor Abemaciclib and HDAC Inhibitor Panobinostat in Pancreatic Cancer Cells

**DOI:** 10.3390/cancers16152713

**Published:** 2024-07-30

**Authors:** Shraddha Bhutkar, Anjali Yadav, Himaxi Patel, Shrikant Barot, Ketan Patel, Vikas V. Dukhande

**Affiliations:** 1 Department of Pharmaceutical Sciences, College of Pharmacy & Health Sciences, St. John’s University, Queens, NY 11439, USA; 2 Department of Oncological Sciences, Icahn School of Medicine at Mount Sinai, New York, NY 10029, USA; 3 The Tisch Cancer Institute, Icahn School of Medicine at Mount Sinai, New York, NY 10029, USA

**Keywords:** abemaciclib, panobinostat, cell cycle, epigenetics, pancreatic cancer, drug synergy

## Abstract

**Simple Summary:**

Pancreatic ductal adenocarcinoma has a low 5-year survival rate due to delayed diagnosis, rapid growth, tumor complexity, and difficulties in surgical resection. Treatments involving new targeted chemotherapeutic options are required due to challenges such as drug resistance, low efficacy, severe toxicity, high metastatic potential, and clinical trial failures. This study demonstrates the preclinical efficacy and potential of the novel combination of abemaciclib and panobinostat in multiple pancreatic cancer cell lines. Our results depict that a novel combination of these agents aids in synergistic effects, causing cell death in pancreatic cancer cells.

**Abstract:**

The current 5-year survival rate of pancreatic cancer is about 12%, making it one of the deadliest malignancies. The rapid metastasis, acquired drug resistance, and poor patient prognosis necessitate better therapeutic strategies for pancreatic ductal adenocarcinoma (PDAC). Multiple studies show that combining chemotherapeutics for solid tumors has been successful. Targeting two distinct emerging hallmarks, such as non-mutational epigenetic changes by panobinostat (Pan) and delayed cell cycle progression by abemaciclib (Abe), inhibits pancreatic cancer growth. HDAC and CDK4/6 inhibitors are effective but are prone to drug resistance and failure as single agents. Therefore, we hypothesized that combining Abe and Pan could synergistically and lethally affect PDAC survival and proliferation. Multiple cell-based assays, enzymatic activity experiments, and flow cytometry experiments were performed to determine the effects of Abe, Pan, and their combination on PDAC cells and human dermal fibroblasts. Western blotting was used to determine the expression of cell cycle, epigenetic, and apoptosis markers. The Abe-Pan combination exhibited excellent efficacy and produced synergistic effects, altering the expression of cell cycle proteins and epigenetic markers. Pan, alone and in combination with Abe, caused apoptosis in pancreatic cancer cells. Abe-Pan co-treatment showed relative safety in normal human dermal fibroblasts. Our novel combination treatment of Abe and Pan shows synergistic effects on PDAC cells. The combination induces apoptosis, shows relative safety, and merits further investigation due to its therapeutic potential in the treatment of PDAC.

## 1. Introduction

Pancreatic ductal adenocarcinoma (PDAC) has one of the highest mortality rates among gastrointestinal malignancies, with a poor prognosis. It accounts for 85–90% of the total pancreatic cancer cases [1]. Pancreatic cancer has a 5-year survival rate of 12% [2,3]. Multi-drug combinations like FOLFIRINOX (5-Fluorouracil, Leucovorin, Irinotecan, and Oxaliplatin) and gemcitabine, along with nab-paclitaxel are the preferred first-line chemotherapeutics for metastatic PDAC [4,5]. However, the long-term use of these drugs can lead to the development of chemoresistance and a further decline in therapeutic efficacy. These issues negatively impact patients’ long-term survival, demanding the development of novel therapies to improve patient outcomes for PDAC [4].

Epigenetic modifications are considered one of the newer hallmarks of cancer progression [6]. These non-mutational changes result in an altered gene expression profile and potentially trigger uncontrolled tumor growth [6]. Much evidence pinpoints the role of histone deacetylase (HDAC) enzymes in the progression of pancreatic cancer by deacetylating histone and non-histone proteins [7]. The post-translational modification facilitated by HDACs results in the transcriptional repression of tumor suppressor genes, including p53, which leads to the progression of the tumor [8,9]. Other evidence suggests that p21, an endogenous inhibitor of cyclin-dependent kinases, is transcriptionally repressed by HDAC4, thereby promoting the growth of colon tumors [10]. SIRT7, a class III HDAC, is reported to be active in the CDKN2A/p16 promoter region, thereby deacetylating H3K18Ac and inhibiting CDKN2A/p16 expression, resulting in cell proliferation in primary lung fibroblasts [11]. Targeting several HDACs represents one of the best-emerging therapeutics for pancreatic cancer. Panobinostat (Pan), a pan-HDAC inhibitor (HDACi), has demonstrated efficacy against pancreatic cancer cells by inducing apoptosis and impeding cell proliferation at nanomolar concentrations [12,13]. The US FDA has approved this drug for refractory multiple myeloma treatment [14]. However, the FDA recently rescinded this approval due to the absence of clinical effectiveness and activity, in contrast to the results observed in hematological malignancies [15]. Several clinical studies have investigated the efficacy and safety of Pan when combined with different chemotherapeutics for solid tumors [16,17].

CDKN2A is one of the most frequently mutated genes (90%) harbored in PDAC patients, resulting in the loss of endogenous p16 encoded by CDKN2A [18,19]. Low levels of cellular p16 promote the phosphorylation of retinoblastoma protein (Rb), leading to S phase entry and subsequent cell cycle progression. Direct intervention in the process of cell cycle progression results from the functional inhibition of CDK4/6 by p16, leading to the hypophosphorylation of Rb and subsequent G0/G1 checkpoint arrest [18]. Multiple direct CDK4/6 inhibitors have been developed and tested in clinical trials for pancreatic cancer. Abemaciclib (Abe) is one of the most potent and selective CDK4/6 inhibitors and is FDA-approved for breast cancer. A recent research study has demonstrated Abe’s efficacy as it induced senescence in both cellular and animal models of pancreatic cancer [20]. Pancreatic cancer is associated with the loss of expression of the endogenous p21 protein, a CDK inhibitor, correlated with the poor overall survival of patients bearing this tumor [21]. In bladder carcinoma, treatment with suberoylanilide hydroxamic acid (SAHA) has been shown to selectively increase the expression of p21, resulting in cell cycle arrest [22]. Similarly, Pan treatment has been shown to elevate the expression of p21 and p27, leading to G0/G1 phase cell cycle arrest in Panc1 cells. It also led to G2/M phase cell cycle arrest, leading to apoptosis in PDAC cells [23].

In most solid tumors, combining chemotherapeutic agents has proven more effective than single agents. Due to drug resistance issues, single-agent treatments show less efficacy than combination treatments [20,24]. Previous studies have shown that inhibiting HDACs affects cell cycle regulation by arresting cells in the G0/G1 phase [12]. These findings highlight the opportunity for a novel and robust combination tackling cell cycle proteins and epigenetic changes, using HDAC and CDK4/6 inhibitors to treat PDAC [25]. Therefore, we hypothesized that combining two different targeted drugs, Abe, a potent CDK4/6 inhibitor, and Pan, a class I, II, and IV HDAC inhibitor, could synergize, producing enhanced anti-cancer effects in PDAC. In this study, we explored the pharmacological effects of the combination of Abe and Pan for treating pancreatic cancer, showing that this combination is highly synergistic and demands further mechanistic and in vivo evaluation.

## 2. Materials and Methods

### 2.1. Chemical Reagents

Abemaciclib, panobinostat, and staurosporin were obtained from MedChemExpress (MCE), Monmouth Junction, NJ, USA; MTT dye (3-(4,5-dimethylthiazol-2-yl)-2,5-diphenyltetrazolium bromide), the CyQUANT™ Direct Cell Proliferation Assay Kit, Invitrogen FxCycle PI/RNase Staining Solution, the Pierce BCA Protein Assay Kit, and the Western blot substrates Pico and Femto were obtained from Thermofisher Scientific, Waltham, MA, USA; Accutase Cell Detachment Solution and FITC Annexin V Apoptosis Detection Kit I were obtained from BD Biosciences, Franklin Lakes, NJ, USA; and the Caspase-3 Fluorometric Assay Kit was obtained from Sigma Aldrich, St. Louis, MO, USA. All primary and secondary antibodies were from Cell Signaling Technology, Danvers, MA, USA, except for Rb, caspase-3, and β-actin, which were from Proteintech, Rosemont, IL, USA.

### 2.2. Cell Culture

Human pancreatic adenocarcinoma cells MIA PaCa-2, BxPC-3, AsPC-1, and normal human dermal fibroblasts (HDF) were obtained from the American Type Culture Collection (ATCC), Manassas, VA, USA. MIA PaCa-2 was maintained aseptically in 25 mM high-glucose Dulbecco’s modified eagle’s medium (DMEM), Corning, Corning, NY, USA. BxPC-3 and AsPC-1 were grown in 11 mM RPMI-1640 media Corning, Corning, NY, USA. Media were supplemented with 10% fetal bovine serum R&D Systems, Minneapolis, MN, USA, and 1% antibiotic-antimycotic Corning, Corning, NY, USA. Human dermal fibroblasts were grown in HDF growth medium Cell Applications, San Diego, CA, USA, along with 1% antibiotic-antimycotic Corning, Corning, NY, USA. All cell lines were maintained aseptically under consistent temperature and humidity control at 37 °C/5% CO_2_.

### 2.3. HDAC and Cell Cycle Gene Expression Analysis in Patient Samples

mRNA expression between normal and tumor patient tissue was determined from the pancreatic adenocarcinoma (PAAD) data set using Gepia2 (Gene Expression Profiling Interactive Analysis) [26]. The same data set was used to populate Kaplan-Meier plots and perform a survival analysis. A cutoff value of quartiles was used to differentiate patients into low- and high-expression groups.

### 2.4. MTT Cytotoxicity Assay

The MTT cell viability assay was utilized to assess the effectiveness of the test compounds in pancreatic cancer cells. This assay is performed by measuring the UV absorbance of formazan crystals formed after the reduction of MTT dye by mitochondrial NADPH-dependent oxidoreductase enzymes. A total of 2000–5000 cells per well were plated and allowed to grow overnight in 96-well plates. The next day, the cells were treated with the indicated concentrations of Abe and Pan for 48 h. Subsequently, 20 µL of MTT dye solution prepared in sterile 1X PBS was added. Cells were incubated with the dye for 3 h at 37 °C/5% CO_2_. Post-incubation, the growth media, including the dye, were aspirated carefully. Dark purple formazan crystals were then dissolved in DMSO, and the absorbance was recorded at 570 nm using the Molecular Devices plate reader SpectraMax M5e. Cellular cytotoxicity (CC_50_) values were computed using non-linear regression analysis using the GraphPad Prism V9 software.

### 2.5. Cell Proliferation Assay

Cell proliferation was measured using the Thermofisher Scientific CyQUANT Direct Cell Proliferation Assay, which consists of a DNA-binding dye and a background quencher. This dye is incorporated into the nuclei of healthy cells and binds to DNA, emitting fluorescence that can be quantified. Pancreatic cancer cells, ranging from 1000 to 2000 cells per well, were seeded in a black clear-bottom fluorescence 96-well plate and allowed to grow overnight. The next day, the cells were treated with individual drugs for 48 h. After the treatment, the nucleic acid stain was aseptically added to the complete growth media in an equal ratio in a 96-well plate containing cells. Cells were incubated with CyQUANT nucleic acid dye for 1 h at 37 °C/5% CO_2_. After incubation with the dye, the fluorescence emitted was measured at certain wavelengths (excitation/emission) maxima 508/527 nm) using a Tecan Spark 10 M multimode microplate reader.

### 2.6. Synergy Study

The synergy between Abe and Pan was assessed using an MTT cytotoxicity assay. Briefly, 2000–5000 cells were seeded in 96 well plates and were allowed to adhere and grow overnight in a 37 °C incubator with 5% CO_2_. The next day, the cells were treated with Abe and Pan alone and in combination for 48 h, and the MTT assay protocol was followed. The % viability data were analyzed using the SynergyFinder software V3.0 [27], and interactive multi-drug synergy plots were downloaded as readouts. Additionally, the synergistic efficacy was determined using the classic Chou-Talalay Combination Index method and its related software, CompuSyn V1.0 [28].

### 2.7. Clonogenic Survival Assay

The proliferative potential of cancer cells can also be measured using the ability of cells to form colonies upon drug insult. MIA PaCa-2 (200 cells/well) and BxPC-3 (1000 cells/well) cells were seeded in 6-well plates and allowed to adhere to the plates in their respective media overnight. The following day, the cells were challenged with individual drugs for 48 h. After treatment, the cells were allowed to grow in drug-free media until the colonies in the control group were sufficiently large. All wells containing the control and treated colonies were fixed in 4% formaldehyde for at least 30 min at 4 °C. After fixation, the colonies were stained with 0.5% crystal violet in methanol for 10 min. The excess stain was washed off, the colonies were allowed to air-dry, and the number of colonies was counted in each well manually. The colony formation ability as a percentage was determined by comparing it with the control.

### 2.8. Cell Cycle Assay

We aimed to quantify the proportion of cells in different cell cycle phases by using a propidium iodide stain. Approximately 0.5 to 1 million MIA PaCa-2 and BxPC-3 cells were plated and allowed to grow overnight in media. The next day, the cells were treated with the respective drugs for 24 h. After treatment, the cells were harvested in PBS and fixed with 70% ethanol overnight at -20 °C. The next day, fixed cells were washed three times with PBS and incubated with FxCycle PI/RNase Staining Solution for 1 h at room temperature in the dark. Following incubation, stained cells were resuspended and analyzed on AMNIS Flowsight using the Ideas software V6.2.

### 2.9. Scratch Assay

The migration behavior of MIA PaCa-2 and BxPC-3 cells upon drug treatment was studied using a scratch assay, as described before [29]. Briefly, 3 × 10^5^ MIA PaCa-2 and 2 × 10^5^ BxPC-3 cells/well were plated in 800 μL media in a 24-well plate. The cells were incubated at 37 °C and 5% CO_2_ overnight. A cell monolayer was allowed to form, and then a scratch was created horizontally in the well using a 200 μL sterile pipette tip. Next, the media in the wells were aspirated, and the cells were washed with PBS to remove any debris. Further, the cells were treated with the respective drug concentrations in 800 μL low-serum media (1% *v*/*v*). Images were taken at several time intervals for MIA PaCa-2 and BxPC-3 cells on an EVOS FL Auto Imaging System Thermofisher Scientific, Waltham, MA, USA. The % gap closure was quantified at five distinct points for the distance between the two lines of the scratch using Adobe Illustrator. The graph was plotted using the GraphPad Prism V9 software.

### 2.10. Invasion Assay

The invasive potential of MIA PaCa-2 and BxPC-3 cells was measured using a transwell migration assay utilizing 24-well plates with inserts (8 μm) from Corning, Corning, NY, USA [30]. A basement membrane was added to the inserts and incubated at 37 °C for 1 h. Next, 24 h serum-starved MIA PaCa-2 and BxPC-3 cells were seeded on the inserts at a density of 50,000 cells/well in serum-free media. The bottom chamber was prepared for the cell invasion assay using 750 μL of 10% FBS in the respective media. MIA PaCa-2 and BxPC-3 cells were treated in a cell migration chamber with the indicated Abe, Pan, and combination concentrations and were incubated at 37 °C/5% CO_2_ for 48 h. The invaded cells were fixed with 4% *v/v* glutaraldehyde solution and stained with 0.5% *w/v* crystal violet. The number of cells that invaded through the basement membrane was counted manually and analyzed using the GraphPad Prism V9 software.

### 2.11. Apoptosis Assay

The sensitive probe FITC Annexin V, which binds to phosphatidylserine, was used to detect the stages of cell death during apoptosis. Approximately 0.5 million MIA PaCa-2 and BxPC-3 cells were plated and grown overnight. The next day, the cells were treated with the drugs for 24 h. After treatment, the cells were collected, washed in PBS, and resuspended in 1X binding buffer. Next, we used the BD FITC Annexin V/PI dual stains and incubated the cells in each dye for 20 min. Proper staining controls were utilized, and the cells were analyzed using AMNIS Flowsight and the Ideas software V6.2.

### 2.12. Caspase-3 Enzyme Activity Assay

We utilized the Sigma Caspase-3 Fluorometric Assay Kit, based on the release of fluorescent 7-amino-4-methylcoumarin (AMC) upon the hydrolysis of Ac-DEVD-AMC by the caspase-3 enzyme. A total of 0.5 million MIA PaCa-2 cells were plated in a cell culture dish and allowed to grow overnight in an incubator. Further, the cells were treated for 24 h with the respective drugs. The treatment was terminated by preparing cell lysates in RIPA buffer (50 mM Tris pH 8.0, 150 mM NaCl, 1% NP40, 0.5% deoxycholate, 0.1% SDS, 10% glycerol, 10 mM NaF, 0.4 mM EDTA) including a protease inhibitor cocktail on ice. Enzyme activity was determined using the manufacturer’s manual. The protein concentration for specific activity was determined using a BCA assay kit.

### 2.13. Western Blotting

Protein expression was analyzed using the Western blotting technique. Around 0.5 million MIA PaCa-2 cells were allowed to grow overnight and treated using the indicated drugs for 24 h. Post-treatment, cells were lysed on ice using RIPA buffer with protease and phosphatase inhibitors with 1 µM trichostatin A (TSA) and 10 mM nicotinamide. The obtained lysates were cold-centrifuged at 10,000 rpm for 10 min. The supernatant was collected in a newly labeled tube, and the protein concentration was determined using a BCA assay kit. Further samples were prepared by adding 4× SDS sample buffer and denaturing them at 95 °C. Proteins were separated on gradient 4–20% SDS PAGE gel and transferred to a methanol-activated polyvinylidene difluoride membrane. Next, the membranes were blocked in 5% milk/BSA in 1X TBST for 1 h and probed with primary antibodies overnight at 4 °C. The next day, the membranes were incubated with a secondary antibody, and protein expression was detected on the Azure c500 Imaging system using a chemiluminescence substrate and further analyzed using the Image J V1.54 software.

### 2.14. Spheroid Assay

MIA PaCa-2 and BxPC-3 cells were seeded at a density of 1500 and 3000 cells per well in ultra-low-attachment 96-well plates, respectively. The cells were incubated at 37 °C/5% CO_2_ for 72 h and 120 h for MIA PaCa-2 and BxPC-3, respectively, to form 3D spheroids. The spheroids were observed every day under the Evos FL2 Auto Imaging System. Next, the MIA PaCa-2 and BxPC-3 spheroids were treated with the designated concentrations of Abe and Pan every 48 h for 10 and 8 days, respectively. Spheroid images were captured every 48 h, and the area was measured using the Evos FL2 Auto Imaging System.

### 2.15. Live-Dead Cell Viability of 3D Tumor Spheroids

The 3D spheroids were analyzed after the designated treatment times with live-dead fluorescent dyes, as described previously [31]. The dyes were prepared for MIA PaCa-2 and BxPC-3 spheroids in their respective media and added to the spheroid plates for 3 h incubation before fluorescent imaging. Calcein AM, Biotium, Fremont, CA, USA was used at a 1 µM concentration to detect live cells, and ethidium homodimer Biotium, Fremont, CA, USA at a 3 µM concentration to detect dead cells. The enzymes acetoxymethyl and intracellular esterase convert the non-fluorescent calcein AM dye into fluorescent calcein upon hydrolysis and emit green fluorescence. In contrast, ethidium homodimer binds to the DNA of dead cells passing through the perforated membrane to emit fluorescence. Live-dead images of the spheroids were taken on the Evos FL2 Auto Imaging System.

### 2.16. Human Dermal Fibroblast Cytotoxicity Assay

Approximately 10,000 cells per well were plated in a 96-well plate and allowed to grow overnight in ambient cell culture conditions. The next day, the cells were treated with Abe and Pan for 48 h. After treatment, the cells were incubated with MTT dye for about 3 h. Post-incubation, the obtained formazan crystals were dissolved in DMSO, and the absorbance was read at 570 nm. Cytotoxicity was calculated using GraphPad Prism V9.

### 2.17. Statistical Analysis

All data reported are the mean ± SEM of a minimum of *n* = 3 individual experiments. For the determination of cellular CC_50_ values, a non-linear regression test was performed on at least three experiments. Bar graph statistics were obtained using ordinary one-way ANOVA or two-way ANOVA on GraphPad V9 software. Within-group comparisons were performed using Dunnett’s or Tukey’s post hoc analysis. *p* values of * *p* < 0.05, ** *p* < 0.01, *** *p* < 0.001 were considered statistically significant.

## 3. Results

### 3.1. HDACs and Cell Cycle Proteins Are Overexpressed in Pancreatic Cancer Patients

**Figure 1:**
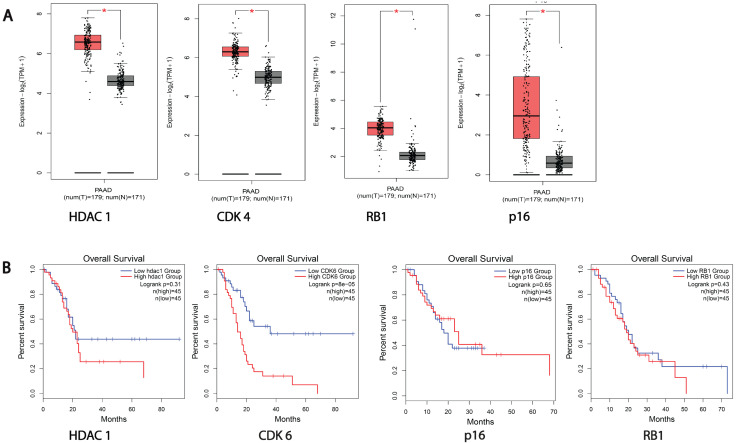
Bioinformatic analysis of epigenetic and cell cycle genes in pancreatic cancer. (**A**) Differential gene expression profiles of epigenetic and cell cycle-related genes in pancreatic cancer patients compared to normal individuals. * denotes significant gene expression difference between normal and cancer patients. (**B**) Kaplan-Meier survival plots of selected genes’ expression in pancreatic cancer patients.

To elucidate the pharmacological targets that are dysfunctional in epigenetics and cell cycle pathways, the gene expression of clinically relevant HDACs and cell cycle gene transcripts was analyzed on the GEPIA2 browser, which uses the TCGA and GTEx databases [26]. The data revealed the upregulation of most HDACs, such as 1, 2, 5, 7, 8, and 9, in pancreatic cancer patient samples compared to normal patients (Figure 1A and Figure S1A). A similar higher expression of the CDK4 and CDK6 genes was seen in PDAC patient samples than in the normal cohort (Figure 1A and Figure S1A). Strikingly, the higher gene expression of tumor suppressors RB1 and CDKN2A/p16 was observed in tumor patients compared to normal patients (Figure 1A). We also performed a Kaplan-Meier survival analysis, which revealed that the higher expression of HDAC1, HDAC2, HDAC7, and HDAC9 led to poor overall survival in pancreatic cancer patients (Figure 1B and Figure S1B). Similarly, the higher expression of the RB1 and CDK6 genes was correlated with poor overall survival in pancreatic cancer patients (Figure 1B). The gene expression and survival analysis of other HDACs, such as HDACs 3, 4, 6, 10, and 11, depicted no significant changes in tumor patients compared to normal patients.

### 3.2. Abe and Pan Decrease Cell Viability and Affect the Proliferation of PDAC Cells

**Figure 2:**
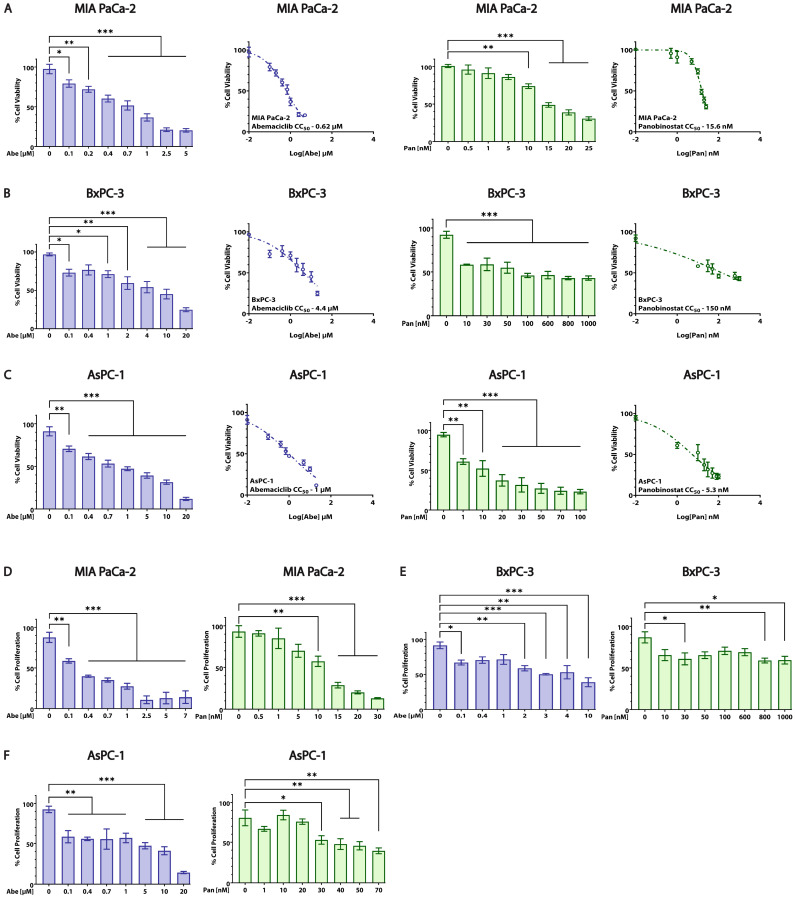
Abe and Pan decrease cell viability and affect the cellular proliferation of pancreatic cancer cells. Cellular viability of (**A**) MIA PaCa-2, (**B**) BxPC-3, and (**C**) AsPC-1 cells upon treatment with Abe and Pan, respectively, for 48 h. One-way ANOVA and post hoc Dunnett test, *n* = 3, * *p* ≤ 0.05, ** *p* ≤ 0.01, *** *p* ≤ 0.001. CC_50_ values for MIA PaCa-2 (**A**), BxPC-3 (**B**), and AsPC-1 (**C**) cells upon treatment with Abe and Pan, respectively, for 48 h, obtained using non-linear regression analysis and log (inhibitor) vs. response curves. The effect of Abe and Pan on PDAC cell proliferation was evaluated in MIA PaCa-2 (**D**), BxPC-3 (**E**), and AsPC-1 cells (**F**), respectively, for 48 h. One-way ANOVA and post hoc Dunnett test, *n* = 3, * *p* ≤ 0.05, ** *p* ≤ 0.01, *** *p* ≤ 0.001.

We utilized an MTT cytotoxicity assay to analyze the effects of Abe and Pan on the viability of pancreatic cancer cells. The dose-response graphs in Figure 2A–C show that Abe significantly decreased the cellular viability of MIA PaCa-2, BxPC-3, and AsPC-1 cells in a dose-dependent manner at 48 h. Similar cytotoxic effects of Pan were observed for all three cell lines with different mutational profiles [32] (Figure 2A–C). As seen in Figure 2A–C and Table 1, both Abe and Pan displayed excellent potency at lower micromolar and nanomolar concentrations across the three studied cell lines based on the log (dose)-response curves and MTT cellular cytotoxicity (CC_50_) values at 48 h. Cancer cells are known for their uncontrolled proliferation and growth properties, which we assessed using the CyQUANT nucleic acid dye. As seen in Figure 2D–F, treatment with Abe and Pan led to a significant decrease in cellular proliferation in all three cell lines at 48 h. In BxPC-3 cells (Figure 2E), Abe- and Pan-treated cells showed a moderate reduction in their cellular proliferation profiles compared to the other two PDAC cell lines at 48 h.

### 3.3. Combined Treatment of Abe and Pan Synergistically Affects the Viability of Pancreatic Cancer Cells

**Figure 3:**
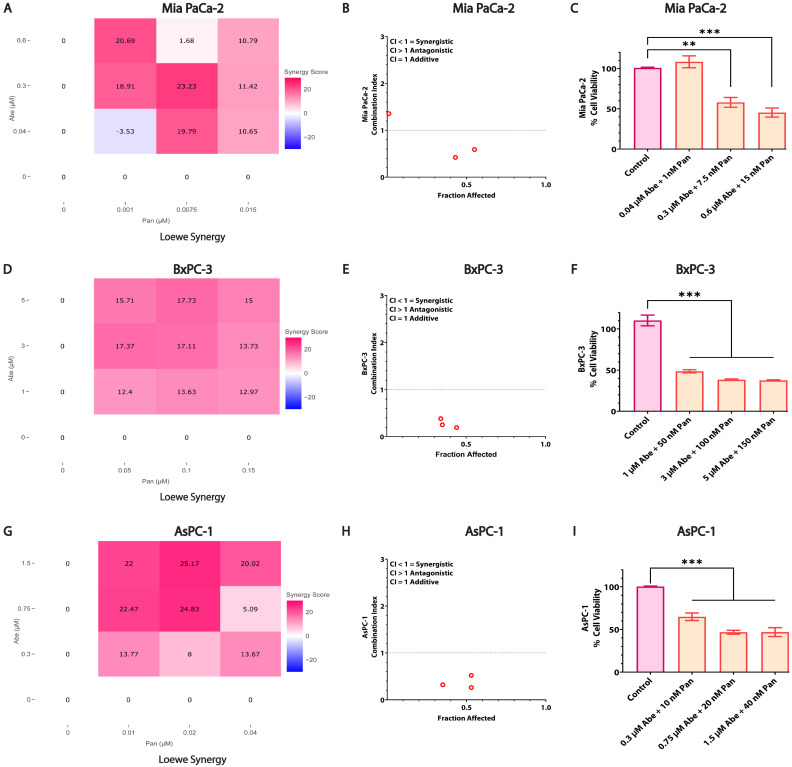
Co-treatment of Abe and Pan in PDAC cells significantly reduces viability. The synergistic efficacy of both Abe and Pan was evaluated in MIA PaCa-2, BxPC-3, and AsPC-1 cells at 48 h using an MTT cell viability assay. (**A**,**D**,**G**) depict the Loewe synergy plots, and (**B**,**E**,**H**) depict the combination index plots for the co-treatment of Abe and Pan in MIA PaCa-2, BxPC-3, and AsPC-1 cells, respectively. Similarly, (**C**) depicts bar graphs for the % cell viability of Abe-Pan co-treated MIA PaCa-2, (**F**) BxPC-3, and (**I**) AsPC-1 cells. One-way ANOVA and post hoc Dunnett test, *n* = 3, ** *p* ≤ 0.01, *** *p* ≤ 0.001.

We performed a cell viability assay to test the combination’s synergy potential by co-administering Abe and Pan to MIA PaCa-2, BxPC-3, and AsPC-1 (Figure 3) cells for 48 h. The SynergyFinder V3.0 and CompuSyn V1.0 software generated interactive synergy plots and graphs depicting the combination indices (CI) against the total fraction affected (FA) by the combined treatments. The lower-concentration co-treatment of both drugs displayed the highest synergy in the Loewe synergy plots for MIA PaCa-2 cells (Figure 3A and Figure S2). A similar trend was observed in BxPC-3 and AsPC-1 cells upon co-treatment with Abe and Pan for 48 h (Figure 3D,G and Figure S2). Chou-Talalay’s CI was calculated using the CompuSyn V1.0 software, which depicted a CI lower than 1, indicating a synergy in the three co-treatments’ FA in all three cell lines (Figure 3B,E,H). Additive or synergistic effects were observed in MIA PaCa-2, BxPC-3, and AsPC-1 cells upon combined treatment, as seen in the CI vs. FA plots (Figure 3B,E,H). The dose-response bar graphs (Figure 3C,F,I) showed a significant reduction in the cell viability of MIA PaCa-2, BxPC-3, and AsPC-1 cells upon treatment with the indicated concentrations of Abe and Pan in combination compared to the control at 48 h.

### 3.4. Abe and Pan Treatment Drastically Affects the Colony Formation Ability of Pancreatic Cancer Cells and Induces Cell Cycle Arrest

**Figure 4:**
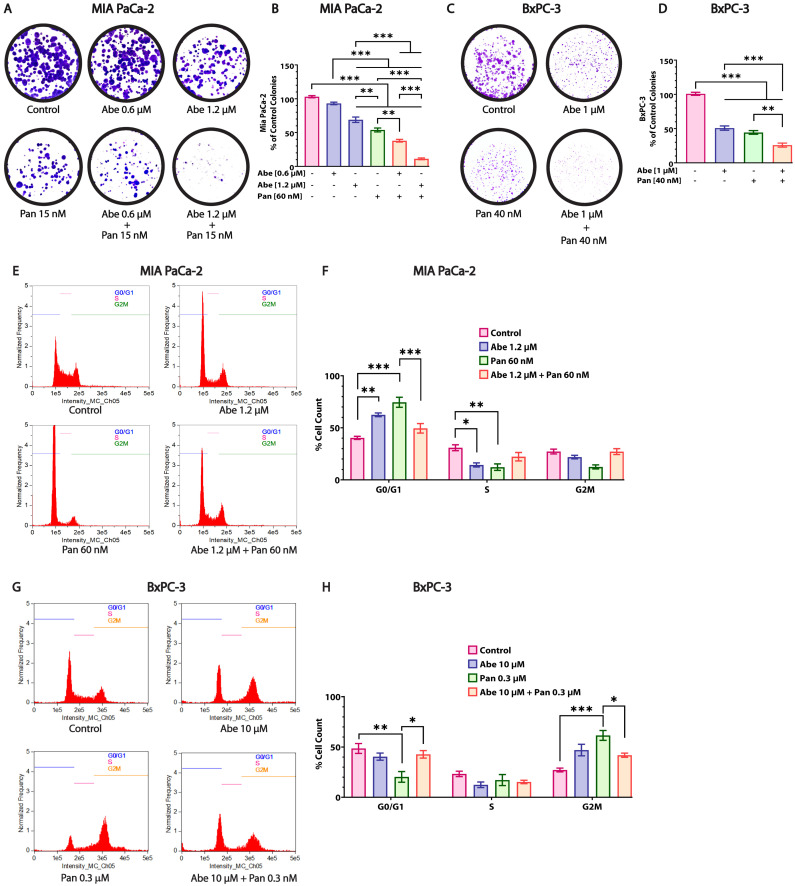
The clonogenic ability and cell cycle of pancreatic cancer cells are severely affected by Abe and Pan treatment. The clonogenic potential of MIA PaCa-2 cells (**A**) and BxPC-3 cells (**C**) was analyzed seven days after individual and combination drug treatments with Abe and Pan for 48 h. The quantification graphs of (**B**) MIA PaCa-2 and (**D**) BxPC-3 colonies. One-way ANOVA and post hoc Tukey’s test, *n* = 3, ** *p* ≤ 0.01, *** *p* ≤ 0.001. Representative data on the cell cycle distribution of (**E**) MIA PaCa-2 and (**G**) BxPC-3 cells upon treatment with the individual drugs and their combination for 24 h; (**F**,**H**) presents their respective quantification. Two-way ANOVA, post hoc Tukey’s test, *n* = 3, * *p* ≤ 0.05, ** *p* ≤ 0.01, *** *p* ≤ 0.001.

The response to chemotherapeutics and the relapse of tumors depend on a drug’s ability to suppress the clonogenic potential of cancer cells. The clonogenic ability of PDAC cells was tested upon drug treatment for 48 h, and sufficient colonies were allowed to form for 7 days until they were countable. Abe-treated cells at a 0.6 µM concentration did not affect the colony formation of MIA PaCa-2 cells (Figure 4A,B). Therefore, we doubled the Abe concentration and observed a substantial reduction in the colonies formed. Pan at 15 nM showed a stronger effect in decreasing the number of colonies of MIA PaCa-2 cells than Abe alone. When cells were treated in combination with Abe and Pan, a drastic and significant reduction in colony formation was observed in MIA PaCa-2 cells compared to the control and individual treatments. In the case of BxPC-3 cells, we utilized lower concentrations of Abe and Pan as the CC_50_ concentrations were extremely detrimental to the cells plated for the clonogenic assay. Similar results were obtained for BxPC-3 cells (Figure 4C,D), as the individual drugs effectively reduced the number of colonies formed, even when treated at lower concentrations than the CC_50_ values, for both Abe and Pan individually at 48 h. Additionally, the combination of Abe and Pan showed a highly synergistic effect in lowering colony formation compared to the individual drugs. The effect of Abe and Pan alone and their combination on the cell cycle phases at 24 h was evaluated in MIA PaCa-2 and BxPC-3 cells by flow cytometry. Therefore, to robustly study the effects of Abe and Pan on the cell cycle process at lower time points, we utilized higher concentrations of both drugs than their respective CC_50_ concentrations in the MIA PaCa-2 and BxPC-3 cell lines. Abe treatment significantly arrested MIA PaCa-2 cells in the G0/G1 phase compared to the control, which is a characteristic feature of CDK4/6 inhibitors (Figure 4E,F). Pan treatment alone also depicted a robust trend by halting MIA PaCa-2 cells in the G0/G1 phase, consistent with the literature (Figure 4E,F) [20]. MIA PaCa-2 cells in the S phase were reduced significantly in both individual drug treatments compared to the control, whereas the combination treatment showed no difference. The percentage of MIA PaCa-2 cells in the G2/M phase was not affected by either of the treatments, including the combined treatment. In the case of MIA PaCa-2 cells, G0/G1 arrest due to Abe and Pan was reduced considerably upon combination treatment, producing no statistically significant effect on the cell cycle pattern. In the case of BxPC-3 cells, the Abe individual treatment and Abe and Pan combination did not change the cell cycle pattern compared to the control. Meanwhile, Pan significantly arrested cells in the G2/M phase compared to the control group (Figure 4G,H). We infer that the effect of the combination was not captured in the cell cycle assay because the combination treatment was severe, resulting in cell death. The dead floating cells were washed away and thereby not captured in certain assays.

### 3.5. Abe and Pan Combination Treatment Inhibited the In Vitro Invasion and Migration of Pancreatic Cancer Cells

**Figure 5:**
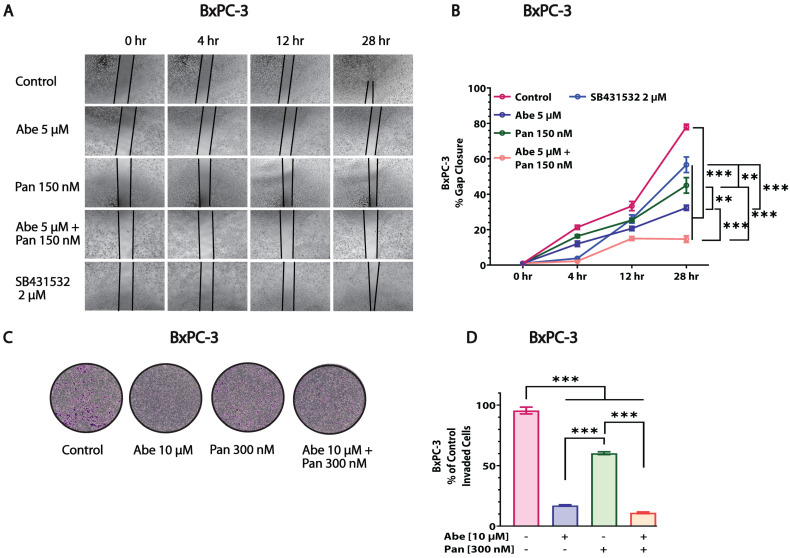
Abe and Pan affect the cellular migratory and invasive abilities when administered individually and in combination in PDAC cells. (**A**) Representative images of the gap closure of the scratch on BxPc-3 cells upon treatment with Abe, Pan, and their combination (scale bar = 1000 µm). (**B**) Graphical representation of the quantification of the gap closure. Two-way ANOVA, *n* = 3, ** *p* ≤ 0.01, *** *p* ≤ 0.001. (**C**) Representative images of the BxPc-3 cells invaded upon treatment with Abe, Pan, and their combination at 48 h. (**D**) Graphical representation of percent cell invasion in BxPC-3 cells. One-way ANOVA, *n* = 3, ** *p* ≤ 0.01, *** *p* ≤ 0.001.

We observed that Abe and Pan’s combination had excellent potential to inhibit the migration of BxPC-3 and MIA PaCa-2 cells compared to the individual drugs using a scratch assay (Figure 5 and Figure S3). Although Pan and Abe individually showed a good effect in terms of inhibiting the migration of both BxPC-3 and MIA PaCa-2 cells, the combination of Abe and Pan was significantly more effective in inhibiting the migration of both cell lines. SB431532, a TGF-β inhibitor, was used as a positive control for the experiment. At the indicated time points, we observed that the drug treatment reduced the scratch’s gap closure, indicating the inhibition of migration in the BxPC-3 and MIA PaCa-2 cells (Figure 5A,B and Figure S3AB). These results support our hypothesis of the enhanced effect of the combination treatment on pancreatic cancer cells compared to independent treatments. Next, we studied the effects of Abe and Pan individually and in combination on the invasive properties of PDAC cells using the transwell migration assay. In BxPC-3 cells, Abe was more effective than Pan when compared to the control (Figure 5C,D). In the case of MIA PaCa-2 cells, Pan demonstrated excellent invasion inhibition compared to the control and Abe alone (Supplementary Figure S3C,D). Additionally, the combination of Abe and Pan treatment significantly reduced the invasion of BxPC-3 and MIA PaCa-2 cells compared to the control and individual drug treatments (Figure 5C,D and Figure S3C,D).

### 3.6. Abe and Pan Treatment Alters the Target Protein Levels in MIA PaCa-2 Cells

**Figure 6:**
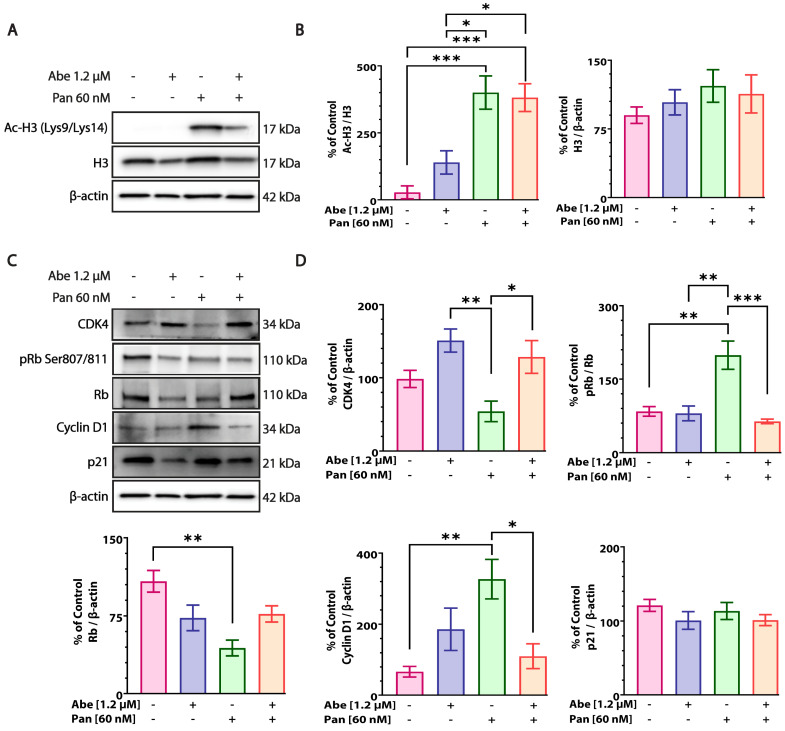
Expression and activity of target proteins upon Abe and Pan treatment. (**A**) The effect of Abe and Pan treatment on the levels of acetylated histone proteins along with co-treatment for 24 h in MIA PaCa-2 cells. (**B**) Quantification of protein levels of Ac-H3 and total H3 upon individual and combined drug treatments. One-way ANOVA and post hoc Tukey’s test, *n* = 4, * *p* ≤ 0.05, *** *p* ≤ 0.001. (**C**) Effects of Abe, Pan, and combination treatment for 24 h on key proteins involved in cell cycle regulation in MIA PaCa-2 cells. (**D**) Graphs of quantitative protein expression of CDK4, Rb, pRb, cyclin D1, and p21 from Western blots. One-way ANOVA and post hoc Tukey’s test, *n* = 4, * *p* ≤ 0.05, ** *p* ≤ 0.01, *** *p* ≤ 0.001.

We performed Western blotting to determine the effects of Abe and Pan treatment on MIA PaCa-2 cells at 24 h, with similar concentrations used as for the cell cycle assay, as both drugs arrested the cells in the G0/G1 cell cycle phase, as reported in the literature. Pan treatment significantly inhibited HDAC enzymes, leading to the accumulation of acetylated histone (Ac-H3), compared to the control and Abe-treated cells, mirroring the results reported in the literature for other HDAC inhibitors [33]. A similar significant increase in the expression of Ac-H3 was observed in the combination-treated cells compared to the control and Abe alone (Figure 6A,B and Figure S5 (replicates related to Figure 6)). We also tested the effects of Abe and Pan alone and in combination on the cell cycle signaling proteins in MIA PaCa-2 cells. Figure 6C,D and Figure S5 (replicates related to Figure 6) show that the Pan treatment reduced the expression of CDK4 as compared with the control and Abe-treated cells. Abe moderately increased the CDK4 protein expression compared to the control group. In the combination-treated cells, the CDK4 expression was higher than in the control and Pan alone. The expression level of total Rb was severely downregulated by the Pan treatment alone compared to the control and Abe-treated cells. A moderate reduction in Rb expression was observed upon Abe treatment alone and combination treatment compared to the control cells. The phospho-Rb (pRb) levels significantly increased upon Pan treatment compared to the control and Abe treatment. The combination treatment depicted a significant reduction in pRb levels compared to Pan alone, suggesting the suppressive effects of Abe treatment on the levels of pRb. The higher expression of cyclin D1 in Pan-treated cells was observed compared to the control and Abe, whose levels declined upon combination treatment. No significant changes in p21 expression were observed under the indicated drug treatments (Figure 6C,D and Figure S5 (replicates related to Figure 6)).

### 3.7. Pan and Abe Combination Treatment Leads to Robust Apoptosis in Pancreatic Cancer Cells

**Figure 7:**
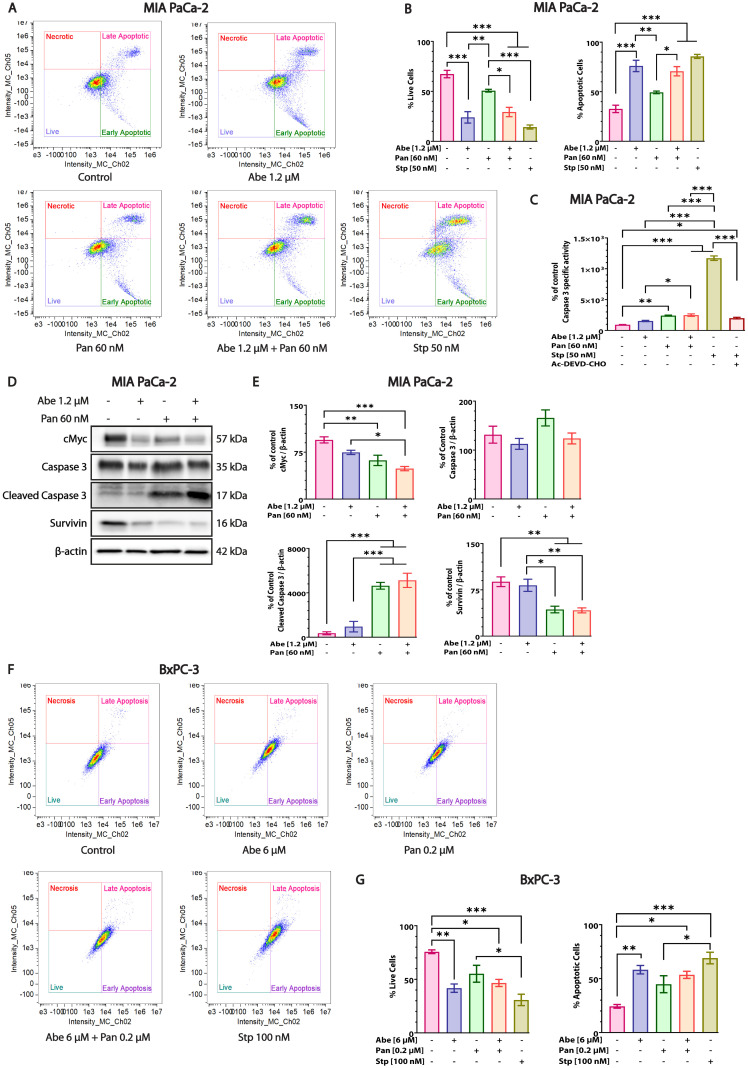
Co-treatment with Abe and Pan induced apoptosis, leading to cell death. (**A**) Annexin V-FITC/PI flow-cytometry-derived dot plots of MIA PaCa-2 and (**F**) BxPC-3 cells upon treatment with indicated concentrations of Abe and Pan for 24 h. Cells were distributed into four quadrants: live, early apoptosis, late apoptosis, and necrosis, based on the Annexin V and PI binding to the cells. Staurosporine (Stp) treatment was used as a positive control. (**B**,**G**) depict quantitative bar graphs of the percentages of live and total apoptotic cells (early + late apoptotic) in MIA PaCa-2 and BxPC-3 cells, respectively. One-way ANOVA and post hoc Tukey’s test, *n* = 3, * *p* ≤ 0.05, ** *p* ≤ 0.01, *** *p* ≤ 0.001. (**C**) Caspase-3 enzyme activity under indicated concentrations of Abe and Pan treatment for 24 h in MIA PaCa-2 cell lysates. One-way ANOVA and post hoc Tukey’s test, *n* = 3, * *p* ≤ 0.05, ** *p* ≤ 0.01, *** *p* ≤ 0.001. Ac-DEVD-CHO: caspase inhibitor. (**D**) Representative Western blots probed for protein markers of cell proliferation, cMyc, and apoptosis markers caspase-3, cleaved caspase-3, and survivin upon treatment with Abe, Pan, and their combination in MIA PaCa-2 cells for 24 h. (**E**) Quantification of Western blots above. One-way ANOVA and post hoc Tukey’s test, *n* = 4, * *p* ≤ 0.05, ** *p* ≤ 0.01, *** *p* ≤ 0.001.

The inhibition of HDAC and CDK4 leads to apoptotic cell death in pancreatic cancer [13,20]. Therefore, we aimed to determine the effect of Abe and Pan alone and in combination on the pancreatic cancer cell death mechanism utilizing similar concentrations for MIA PaCa-2 cells as in the cell cycle assay at 24 h. The flow-cytometric analysis of MIA PaCa-2 and BxPC-3 cells revealed that Abe and Pan treatment induced a reduction in the percentage of live cells compared to the control group, which was further reduced upon combination treatment (Figure 7A,B,F,G and Figure S4). Consequently, the percentage of total apoptotic cells increased in both the individual-drug-treated and co-treated cells. In addition, the activity of the caspase-3 enzyme, a critical player in the apoptosis cascade, was evaluated, and staurosporine (stp) was used as an apoptosis inducer. Pan-treated MIA PaCa-2 cells exhibited a significant increase in caspase-3 activity compared to the control and Abe-treated MIA PaCa-2 cells (Figure 7C). Similar results were observed in the Abe and Pan co-treated cells. Abe-treated cells did not show an increase in caspase-3 enzymatic activity (Figure 7C). We also investigated the effects of Abe and Pan on the protein expression of cMyc, a tumor oncogene overexpressed in pancreatic cancer. Pan-treated and combination-treated cells depicted a significant decrease in cMyc protein levels in the MIA PaCa-2 cell line (Figure 7D,E). Next, the protein expression levels of caspase-3 and cleaved caspase-3 were determined in MIA PaCa-2 cells (Figure 7D,E and Figure S5 (replicates related to Figure 7)). The caspase-3 protein expression remained relatively unaltered in all treatment groups in the MIA PaCa-2 cells. Further, we checked the expression levels of cleaved caspase-3, an active form of executioner caspase-3 causing apoptosis. Abe alone did not increase the cleaved caspase-3 levels compared to the control. However, the Pan treatment and the combination treatment significantly increased the cleaved caspase-3 protein levels compared with the control and Abe-treated MIA PaCa-2 cells. Next, we examined the expression of the survivin protein, an inhibitor of the apoptosis protein (IAP), upon individual drug and combination treatment in pancreatic cancer cells. The survivin protein expression drastically decreased upon Abe and Pan treatment and combination treatment compared with the control MIA PaCa-2 cells (Figure 7D,E and Figure S5 (replicates related to Figure 7)).

### 3.8. Abe and Pan Treatment Severely Reduces the 3D Spheroid Growth of PDAC Cells and Moderately Affects Human Dermal Fibroblast (HDF) Cell Viability

**Figure 8:**
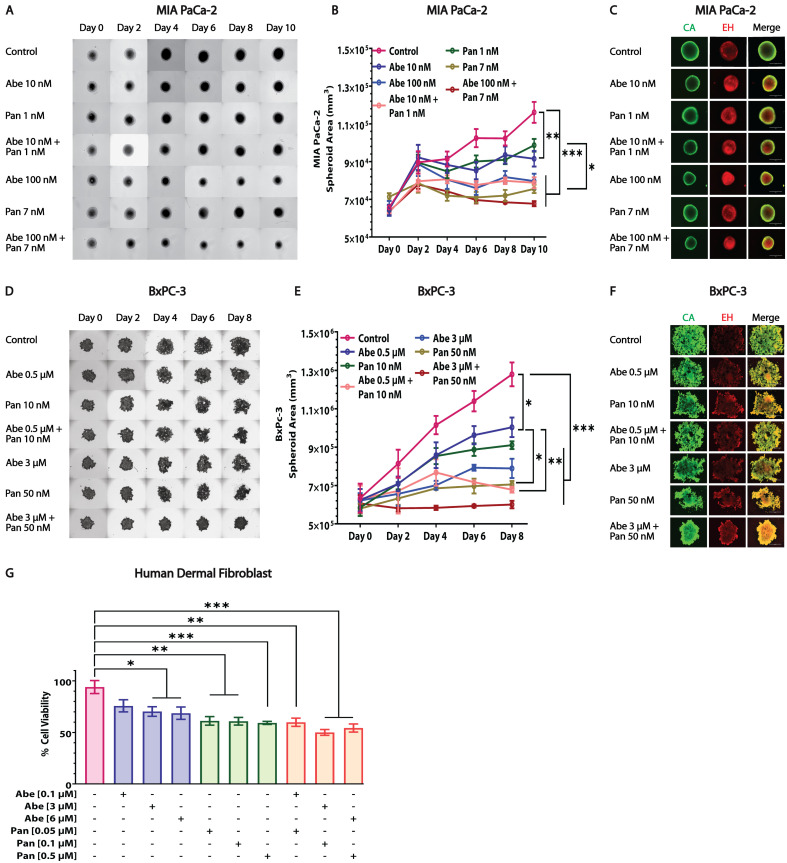
Abe and Pan treatment adversely affects the 3D spheroid growth of PDAC cells and shows lower cytotoxicity against human dermal fibroblast cells. (**A**) Representative brightfield images of MIA PaCa-2 and (**D**) BxPC-3 spheroids treated with Abe and Pan at indicated concentrations every 48 h. Scale bar: 1000 µm. The quantification of the area of spheroids for (**B**) MIA PaCa-2 and (**E**) BxPC-3 spheroids. Two-way ANOVA, post hoc Tukey’s test, *n* = 3, * *p* ≤ 0.05, ** *p* ≤ 0.01, *** *p* ≤ 0.001. Fluorescent images for (**C**) MIA PaCa-2 and (**F**) BxPC-3 spheroids upon indicated Abe and Pan treatment. CA: calcein AM, EH: ethidium homodimer. Scale bar: 400 µm. (**G**) Quantified graph of cell viability of Abe- and Pan-treated HDF cells alone and in combination for 48 h. One-way ANOVA and post hoc Dunnett test, *n* = 3, * *p* ≤ 0.05, ** *p* ≤ 0.01, *** *p* ≤ 0.001.

The efficacy of Abe and Pan treatment was measured in the MIA PaCa-2 and BxPC-3 3D spheroid model. Our results indicate that Abe and Pan are individually effective drugs for both PDAC cell lines (Figure 8A–F). In addition, the Abe and Pan combination, even at lower doses, showed a highly synergistic effect in inhibiting the growth of 3D spheroids. In Figure 8A,D, the brightfield images show the reduced growth of spheroids treated with the Abe and Pan combination compared to the control and individually treated groups. Increased red and reduced green cellular fluorescence was observed upon combination treatment, indicating more dead cells in the spheroids in the combination-treated group (Figure 8C,F). We also studied the toxicity of the Abe and Pan treatments alone and in combination on normal HDF for 48 h. Our results indicated that the combination-treated cells (Figure 8G) showed reduced cell viability compared to the single treatments. However, the concentrations of Abe and Pan used in this assay were significantly higher than the working concentrations used for the treatment of pancreatic cancer cells and produced a much smaller decrease in viability in HDF compared to PDAC cells, suggesting the relative safety of Abe and Pan treatments in HDF.

## 4. Discussion

Cell cycle and epigenetic alterations are hallmarks of cancer growth and proliferation. Therefore, we focused on targeting both aspects in our study. We studied the effects of the combination of Abe, a CDK4/CDK6 inhibitor, and Pan, a pan-HDAC inhibitor (HDACi), on pancreatic cancer cells. Our cell viability and proliferation data suggest that combining Abe and Pan is more effective in killing pancreatic cancer cells than individual treatments. Moreover, apoptosis and western blot experiments showed that the combination treatment targeted the cell cycle pathway and epigenetic alterations to induce apoptosis in pancreatic cancer cells. Our research reveals the pharmacological effects of the novel therapeutic combination of Abe and Pan in pancreatic cancer.

The poor prognosis and mere 12% 5-year survival rate require the scientific community to develop and test novel therapeutics for PDAC [2]. Mutations in oncogenic KRAS and tumor suppressors TP53, SMAD4, and CDKN2A are hallmarks of PDAC, leading to tumor progression and metastasis [34]. Global epigenetic alterations are considered drivers of tumorigenesis, cell cycle processes, tumor cell proliferation, metastasis, etc., leading to cancer progression [35]. Therefore, these non-mutational modifications are now considered one of the hallmarks of cancer [6]. Major post-translational modifications, such as the frequent methylation of 5′ cytosines of DNA by DNMTs or the removal of acetyl marks on histone tails by HDACs, lead to alterations in the expression of tumor suppressor genes, oncogenes, and key signaling and metabolic proteins fueling tumor growth [36]. HDACs exhibit distinct molecular mechanisms regulating tumor growth and proliferation, cancer metabolism, invasion and metastasis, cell cycle control, and cell death processes [37]. Similar to our gene expression analysis data from patient samples (Figure 1 and Figure S1), the overexpression of HDACs has been reported and targeted by HDACi in several other malignancies, including PDAC [38,39]. Notably, HDAC1-positive tumors have been correlated with pancreatic cancer patients’ low survival compared to HDAC1-negative tumors [40]. Our study shows the dose-dependent efficacy of the pan-HDACi Pan in reducing cell viability and the proliferation of PDAC cell lines of different mutational status (Figure 2). The aberrant activation of the cyclin-dependent kinase 4/cyclin D1 pathway is reported frequently in pancreatic cancer due to the overexpression of D-type cyclin proteins [41]. Moreover, the mutational inactivation of tumor suppressor CDKN2A encoding p16 causes the suppression of Rb dependent-G1 arrest, leading to uncontrolled cell cycle progression in PDAC [20]. Drugs targeting CDK4/6 kinases phenocopying p16 are in use for breast cancer and are being tested for PDAC [20,42]. Consistent with previous studies, we observed that Abe treatment effectively reduced cell viability and proliferation in pancreatic cancer (Figure 2).

Most chemotherapeutic drugs show signs of intrinsic or acquired resistance when used against pancreatic cancer. Recent studies have indicated that the effectiveness of CDK4/6 inhibitors is challenged due to acquired resistance in breast cancer patients [43]. Similarly, drug resistance is a key issue for HDACi, where these drugs have failed in the treatment of solid tumors in clinical trials as single agents [44]. The increased expression of anti-apoptotic proteins such as Bcl-2 may confer HDACi resistance [24]. Another piece of evidence shows that the HDACi-mediated upregulation of p21 decreases the cytotoxic effects of these inhibitors in vitro [45]. Additionally, butyrate-resistant HeLa clones have increased proliferation, expressing higher levels of p21 and cyclin D1 [46]. The most effective therapy for PDAC at present is a multi-drug regimen of gemcitabine, nab-paclitaxel, and FOLFIRINOX, suggesting the higher efficacy potential of combination strategies [47]. In vitro studies indicate that Pan is effective in pancreatic cancer when used with other drugs, such as PI3K inhibitors [23], CHK1 inhibitors [12], mTOR inhibitors [48], etc. The combined effectiveness of CDK4/6 inhibitors has also been tested in PDAC along with HuR/YAP inhibitors [20], mTOR inhibitors [42], ERK inhibitors [49], etc. However, the combination of the CDK4/6 inhibitor Abe with the HDACi Pan has not been studied or reported thus far in PDAC cells. In a recent study, a CRISPR-CAS9 loss of function screen revealed a spectrum of functional genes whose inhibition might complement the tumor inhibition properties of CDK4/6 inhibitors in PDAC, in which HDAC genes were identified as positive hits [49]. Our study shows, for the first time, that the pharmacologically targeted combination of Abe and Pan is synergistic and effective in killing pancreatic cancer cells, as depicted by the clonogenic potential (Figure 4), combination synergy studies (Figure 3 and Figure S2), and the live-dead assay of 3D spheroids and spheroid growth studies (Figure 8). We also show that this combination is relatively non-toxic at its effective concentrations on HDF cells, revealing its potential for clinical use (Figure 8).

HDACi halt tumor cell proliferation and growth by disrupting the G0/G1 and G2/M phases of the cell cycle [12,33]. CDK4/6 inhibitors are also reported to cause the G1 arrest of cancer cells in PDAC, leading to senescence and cell death [20]. When treated individually, our flow cytometry data revealed that both drugs, Abe and Pan, arrested pancreatic cancer cells in the cell cycle’s G0/G1 or G2/M phase (Figure 4), similar to previous reports [20,23,33]. Pan-HDACi like Pan are reported to induce the expression of endogenous cell cycle regulator p21, an endogenous cell cycle inhibitor in PDAC, leading to G0/G1 arrest [23]. In our study, we observed a similar increase in the protein expression of p21 in Pan-treated MIA PaCa-2 cells, confirming their G0/G1 arrest (Figure 6C,D and Figure S5 (replicates related to Figure 6)). HDACi are known to downregulate the expression of CDKs [50]. Our study confirmed this finding, where CDK4 expression was downregulated upon Pan treatment in PDAC cells (Figure 6C,D and Figure S5 (replicates related to Figure 6)). Previous reports have suggested that HDACi decrease the phospho-Rb (pRb) levels in vascular smooth muscle cells [51] and cancer cells [52,53]. We assessed the protein expression levels of total Rb and pRb (Ser 807/811) in Pan-treated pancreatic cancer cells. Surprisingly, we observed the significant upregulation of the pRb levels in MIA PaCa-2 cells. We also observed the severe downregulation of the total Rb protein expression levels in Pan-treated cells (Figure 6C,D and Figure S5 (replicates related to Figure 6)), consistent with the downregulated gene expression of RB1 in human breast cancer cells [54]. An HDACi-mediated decrease in cyclin D1 expression is reported in multiple solid tumors, including PDAC [33,55]. However, some reports suggest that the expression of cyclin D1 is upregulated upon treatment with HDACi like valproic acid in human melanoma cells [56], butyrate in vascular smooth muscle cells [51,57,58], MHY219 in prostate cancer cells [59], SAHA in rhabdoid tumors [60], and valproic acid in renal cell carcinoma [61] and lung cancer cells [62]. We observed the significant upregulation of cyclin D1 protein expression in Pan-treated cells (Figure 6C,D and Figure S5 (replicates related to Figure 6)), suggesting that HDACi’ effects on cell cycle proteins are complex and context-dependent. Earlier studies in breast cancer have shown that CDK4/6-inhibitor-treated cells depict early adaptive responses and quickly evade cytostasis by amplifying the cyclin D1-CDK2 axis [63,64]. CDK4 and cyclin D1 overexpression has been reported to cause resistance to CDK4/6 inhibitors in breast cancer cells [65]. A similar adaptive overexpression of cyclin D1 was observed upon treatment with palbociclib in pancreatic cancer cells [66]. Additionally, the co-deletion of CDKN2A and CDKN2C has been reported to determine the sensitivity of CDK4/6 inhibitors in GBM [67]. We observed similar effects in Abe-treated MIA PaCa-2 cells, such as upregulated CDK4 protein expression, slightly downregulated Rb expression, slightly upregulated cyclin D1 expression, and no effect on pRb and p21 protein expression (Figure 6C,D). These results suggest that CDK4 inhibitors alone might not be the best treatment strategy for metastatic cancers like PDAC. Therefore, we attempted to test the combination of Abe and Pan to target the pathway deregulation associated with cell cycle dysfunction and epigenetic alterations in pancreatic cancer cells. Combination therapies with HDACi and cell cycle inhibitors have been tested in previous studies in liver cancer [68], breast cancer [25], lung cancer [62], and malignant ascites [69]. In our study, upon testing the combination of Abe and Pan in MIA PaCa-2 cells in the propidium iodide cell cycle assay, little difference was observed between the control and combination groups, hinting that common mechanistic processes may be involved in their effects on the cell cycle (Figure 4). We also saw increased CDK4 and Rb expression in the combination treatment compared to the single-agent treatments (Figure 6C,D and Figure S5 (replicates related to Figure 6)). These changes may be adaptive in nature. Interestingly, the combination treatment showed the desired profile for pRb, cyclin D1, and Ac-H3K9/K14, which highlights the beneficial effects of the combination.

The invasion and metastasis of cancer cells initiate with epithelial to mesenchymal transition (EMT) processes and together constitute one of the hallmarks of cancer [6]. HDACs play an active role in the invasive and metastatic potential of solid cancers such as renal cell carcinoma [70] and breast cancer [71]. Pan showed excellent potential in inhibiting invasion and metastasis in hepatocellular carcinoma [72], similar to our data in pancreatic cancer cells (Figure 5 and Figure S3). However, the effect of Abe on invasion and metastasis is context-dependent. Resistance to Abe is associated with enhanced metastatic potential in murine breast cancer cells [73]. Similarly, in pancreatic cancer [74], Abe’s treatment induced an increase in the invasive and metastatic potential of cancer cells. However, acute treatment with Abe is reported to decrease the migration of thyroid cancer cells [75]. Our invasion and migration data (Figure 5 and Figure S3) demonstrate the significant effects of Abe in lowering invasion and migration in BxPC-3 cells and migration in MIA PaCa-2 cells. However, the effect of Abe on invasion was modest in MIA PaCa-2 cells. Our study shows the excellent inhibitory effects on invasion and migration for the Abe and Pan combination in both MIA PaCA-2 and BxPC-3 cells and thereby provides a rationale for the use of this combination therapy to treat pancreatic cancer.

Apoptosis is a cell death process by which most chemotherapeutics induce cell death and affect tumor growth [76]. HDACi like valproic acid and trichostatin A are known to cause apoptosis through the intrinsic pathway by disrupting the mitochondrial membrane potential in pancreatic cancer cells [77]. Pan has previously been reported to induce apoptosis in pancreatic cancer cells by caspase-3 activation [13]. In our study, Pan showed an increased percentage of apoptotic cells compared to the control in MIA PaCa-2 and BxPC-3 cells (Figure 7A,B,F,G). We also showed that Pan lowered the cMyc protein expression levels, suggesting a Myc-dependent decrease in proliferation upon HDACi (Figure 7D,E and Figure S5 (replicates related to Figure 7)), as reported previously in pancreatic cancer cells [78]. The flow-cytometric analysis of Abe-treated cells showed the upregulation of the apoptotic cell population (Figure 7A,B,F,G) in MIA PaCa-2 and BxPC-3 cells. However, no effect was observed upon Abe treatment on the protein activity and expression levels of apoptotic proteins like cleaved caspase-3 (Figure 7C–E and Figure S5 (replicates related to Figure 7)), in contrast with earlier data that depict the apoptotic potential of Abe in PDAC cells [20]. We also showed the downregulation of survivin protein expression in Abe-treated cells, as reported previously in lung cancer cells upon treatment with palbociclib [79]. Therefore, apoptosis may play a part, but other processes may be involved in the Abe-induced death of PDAC cells. We also observed the increased depletion of cMyc protein expression in Abe-treated MIA PaCa-2 cells, suggesting that Abe affects the proliferation of cancer cells (Figure 7D,E and Figure S5 (replicates related to Figure 7)), as reported previously for palbociclib in PDAC [80]. In our study, we also showed that when administered in combination, Abe and Pan synergistically induced apoptosis and caused cell death in pancreatic cancer cells (Figure 7 and Figure S4). The effect of Abe and Pan’s combination must be further studied in terms of mechanistic details and cell death processes.

## 5. Conclusions

Our novel study highlights the synergistic therapeutic potential of the novel combination of Abe, a CDK4/6 inhibitor, and Pan, an HDACi when tested in vitro in highly aggressive pancreatic cancer cells. Herein, we show that, when administered individually and in combination, Abe and Pan affect pancreatic cancer cells by altering cell cycle pathway protein expression and inhibiting histone protein deacetylation, ultimately causing cell death. Finally, we showed that the combination treatment of Abe and Pan led to apoptosis and inhibited migration and invasion in pancreatic cancer cells. The 3D spheroids of PDAC cells were drastically inhibited by the combination of Abe and Pan. In addition, we showed that this combination was safe when tested on normal human cells. Future mechanistic and in vivo studies are warranted to decipher how the combination of both Abe and Pan exerts anti-cancer effects in pancreatic cancer.

## Figures and Tables

**Table 1:** CC_50_ values of Abe and Pan on PDAC cell lines along with their mutation status.

Cell Line	Abemaciclib CC_50_ ± SEM (µM)	Panobinostat CC_50_ ± SEM (nM)
MIA PaCa-2 (KRAS-mut, p16-mut, p53-mut)	0.62 ± 0.059	15.6 ± 0.785
BxPC-3 (KRAS-wt, p16-mut, p53-mut)	4.4 ± 2.168	150 ± 0.204
AsPC-1 (KRAS-mut, p16-mut, p53-mut)	1 ± 0.251	5.3 ± 0.783

## Data Availability

The original contributions presented in the study are included in the article/Supplementary Materials; further inquiries can be directed to the corresponding author.

## Supplementary Materials

The following supporting information can be downloaded at https://www.mdpi.com/article/10.3390/cancers16152713/s1, Figure S1: (A) Gene expression profiles of HDAC and cell-cycle-related genes of PDAC patients and normal individuals. (B) Kaplan-Meier plots of differentially expressed genes in pancreatic cancer patients; Figure S2: Bliss synergy plots for (A) MIA PaCa-2, (B) BxPC-3, and (C) AsPC-1 cells, depicting the synergistic potential of Abe and Pan in combination at 48 h; Figure S3: Brightfield images of (A) MIA PaCa-2 cells upon Abe and Pan treatment and their combination for migration assay and (C) invasion assay. (B) and (D) represent quantification graphs for the same; Figure S4: Brightfield images of MIA PaCa-2 cells upon indicated treatments for 24 h. Scale bar = 1000 µm; Figure S5: Raw Western blot images related to Figure 6 and Figure 7 (replicates 1, 2, 3, and 4, respectively).
