# Supplementary figures and images for: Synergistic Efficacy of CDK4/6 Inhibitor Abemaciclib and HDAC Inhibitor Panobinostat in Pancreatic Cancer Cells
Source: Cancers (Basel). 2024 Jul 30;16(15):2713. doi: 10.3390/cancers16152713 (PMC11311278; doi:10.3390/cancers16152713)

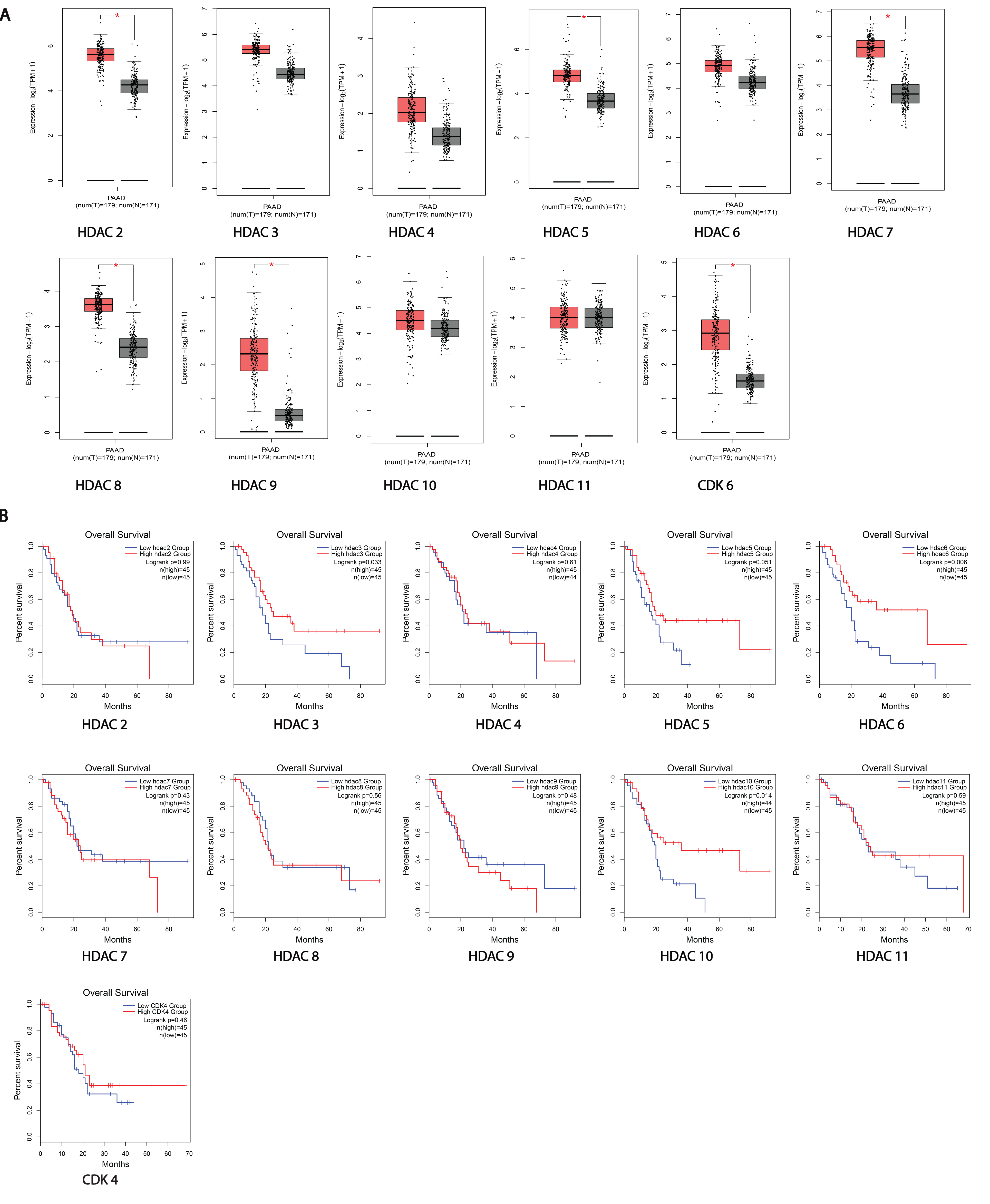

Supplement: Supplementary file 1 [file cancers-16-02713-s001.zip › Figure S1. Bioinformatics Sup-01.tif]

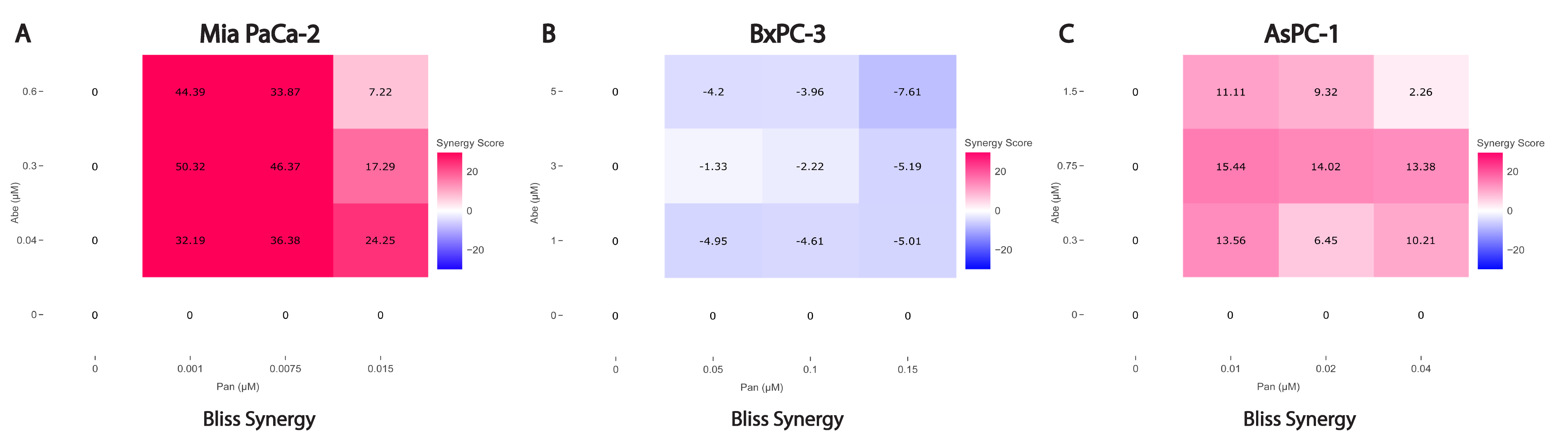

Supplement: Supplementary file 1 [file cancers-16-02713-s001.zip › Figure S2. Combination Studies Sup-02.tif]

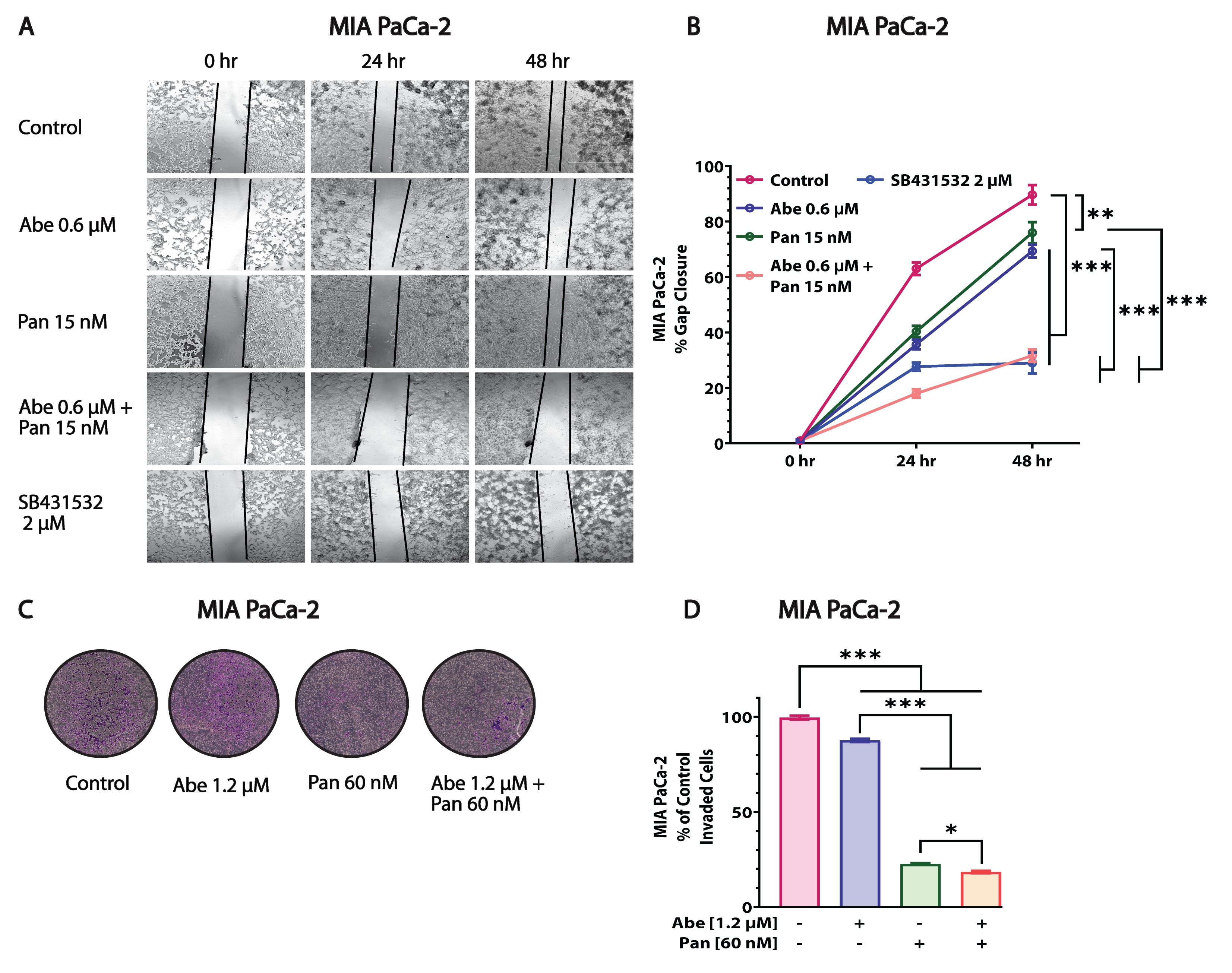

Supplement: Supplementary file 1 [file cancers-16-02713-s001.zip › Figure S3. Invasion and scratch Sup-03.tif]

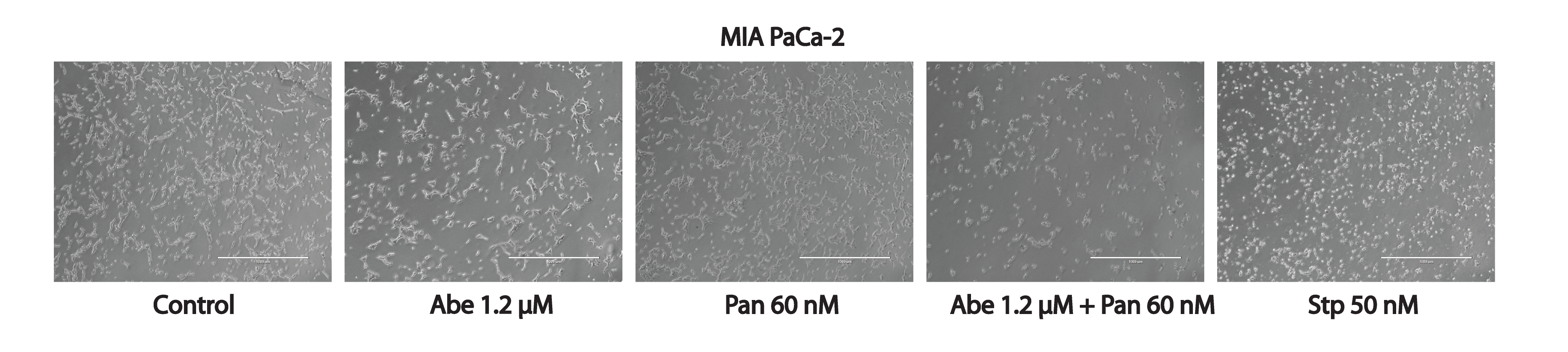

Supplement: Supplementary file 1 [file cancers-16-02713-s001.zip › Figure S4. Evos Images Sup-04.tif]

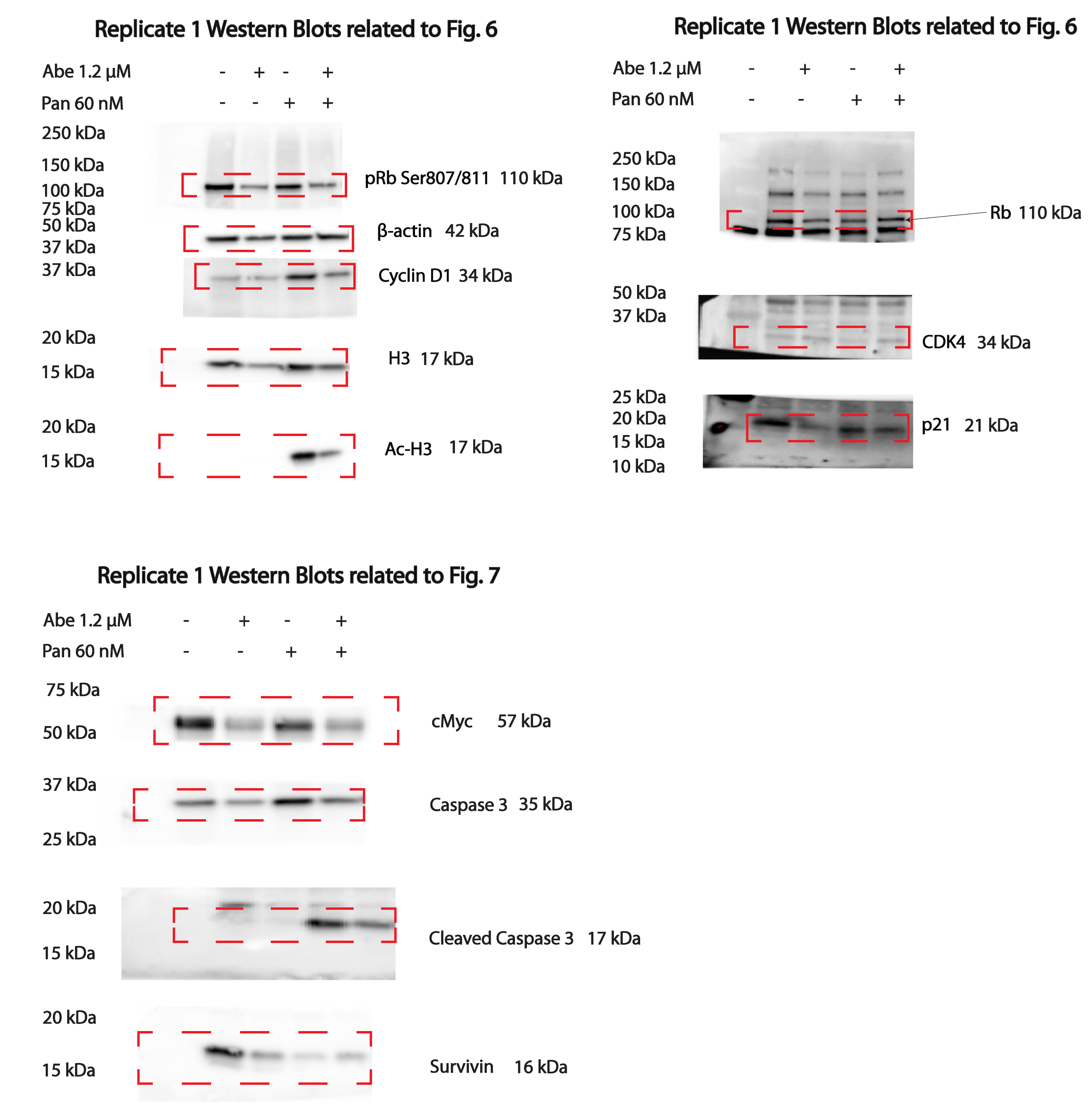

Supplement: Supplementary file 1 [file cancers-16-02713-s001.zip › Figure S5 Raw Western Blots Sup - Rep 1.tif]

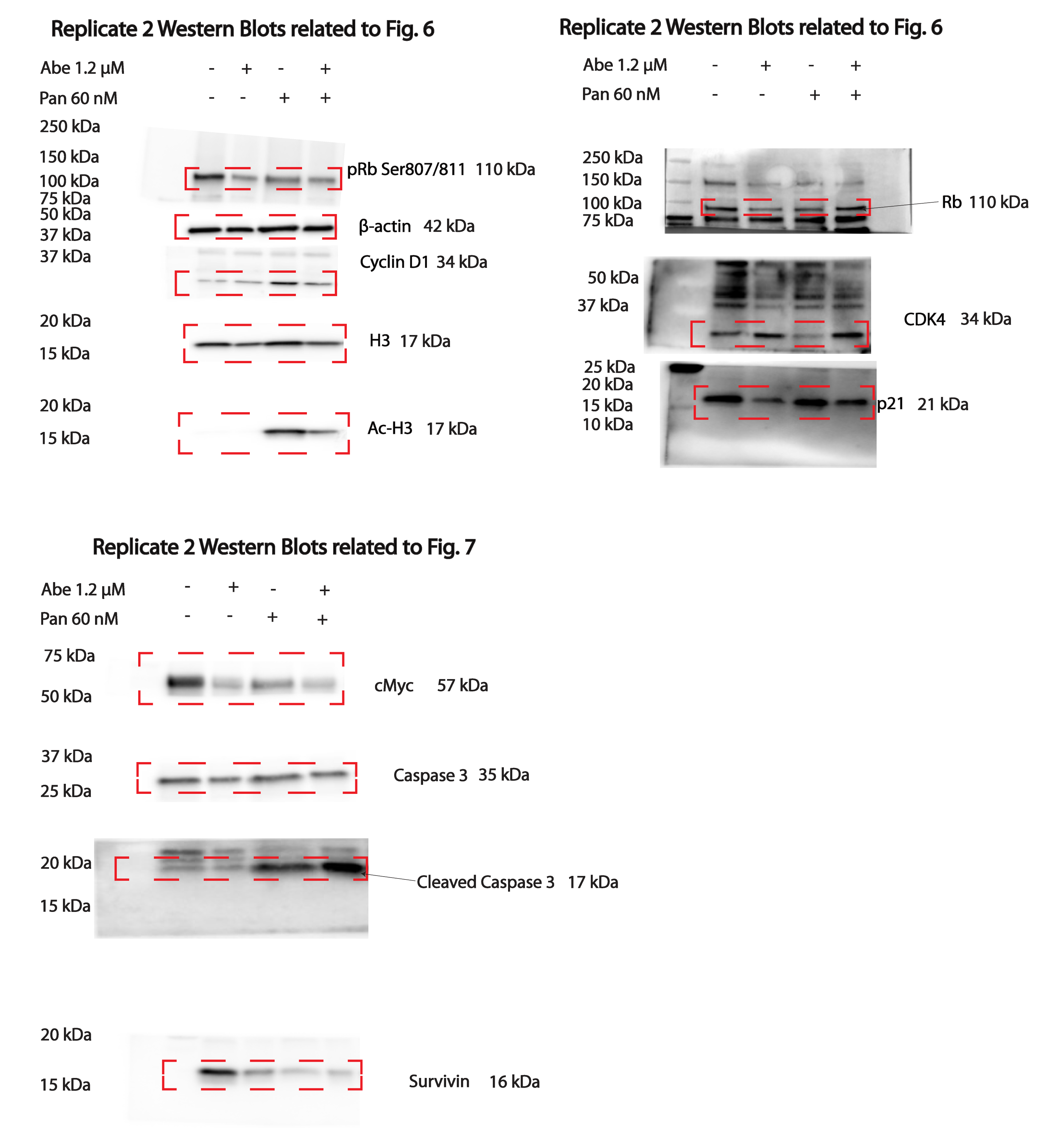

Supplement: Supplementary file 1 [file cancers-16-02713-s001.zip › Figure S5 Raw Western Blots Sup - Rep 2.tif]

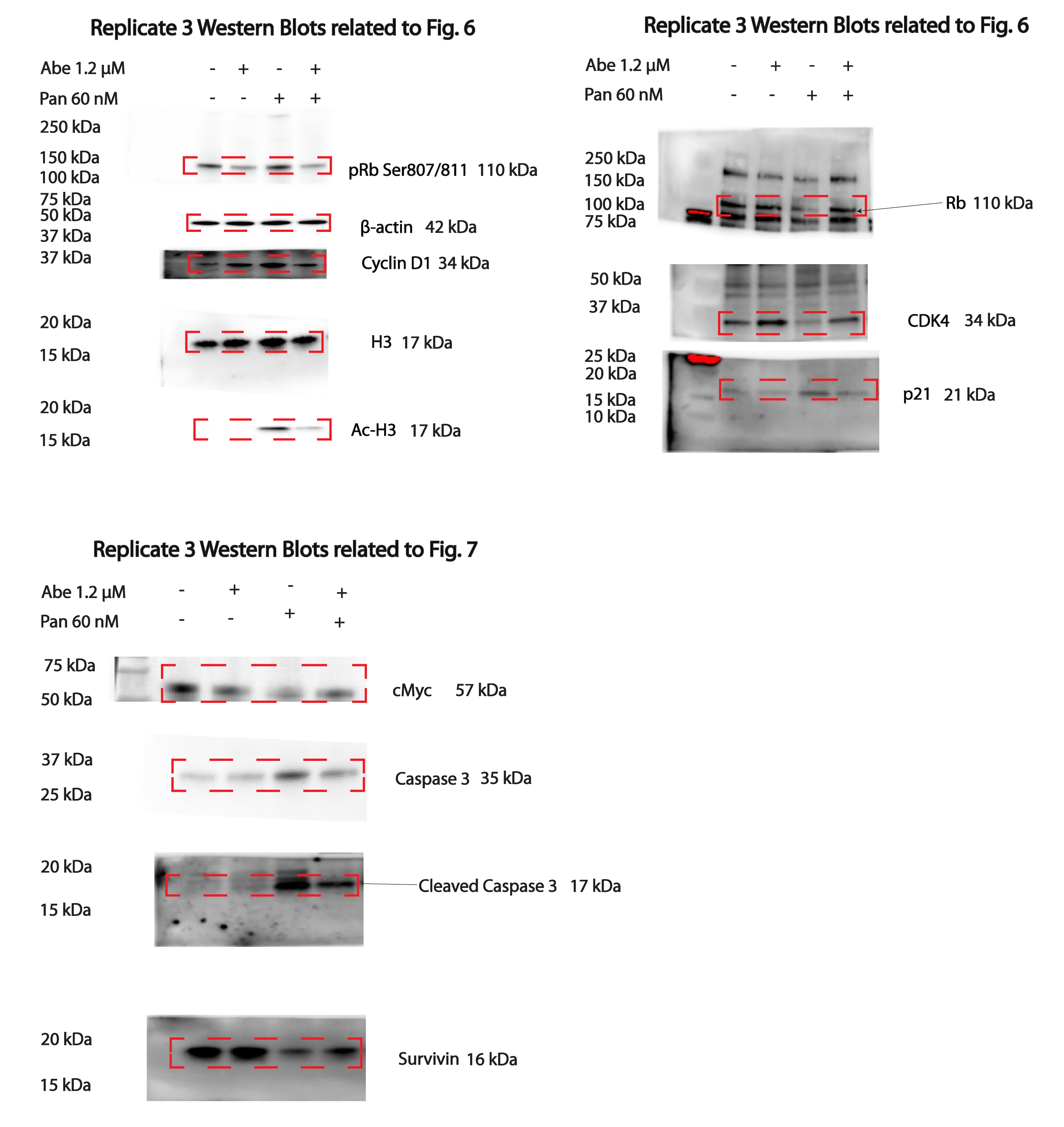

Supplement: Supplementary file 1 [file cancers-16-02713-s001.zip › Figure S5 Raw Western Blots Sup - Rep 3.tif]

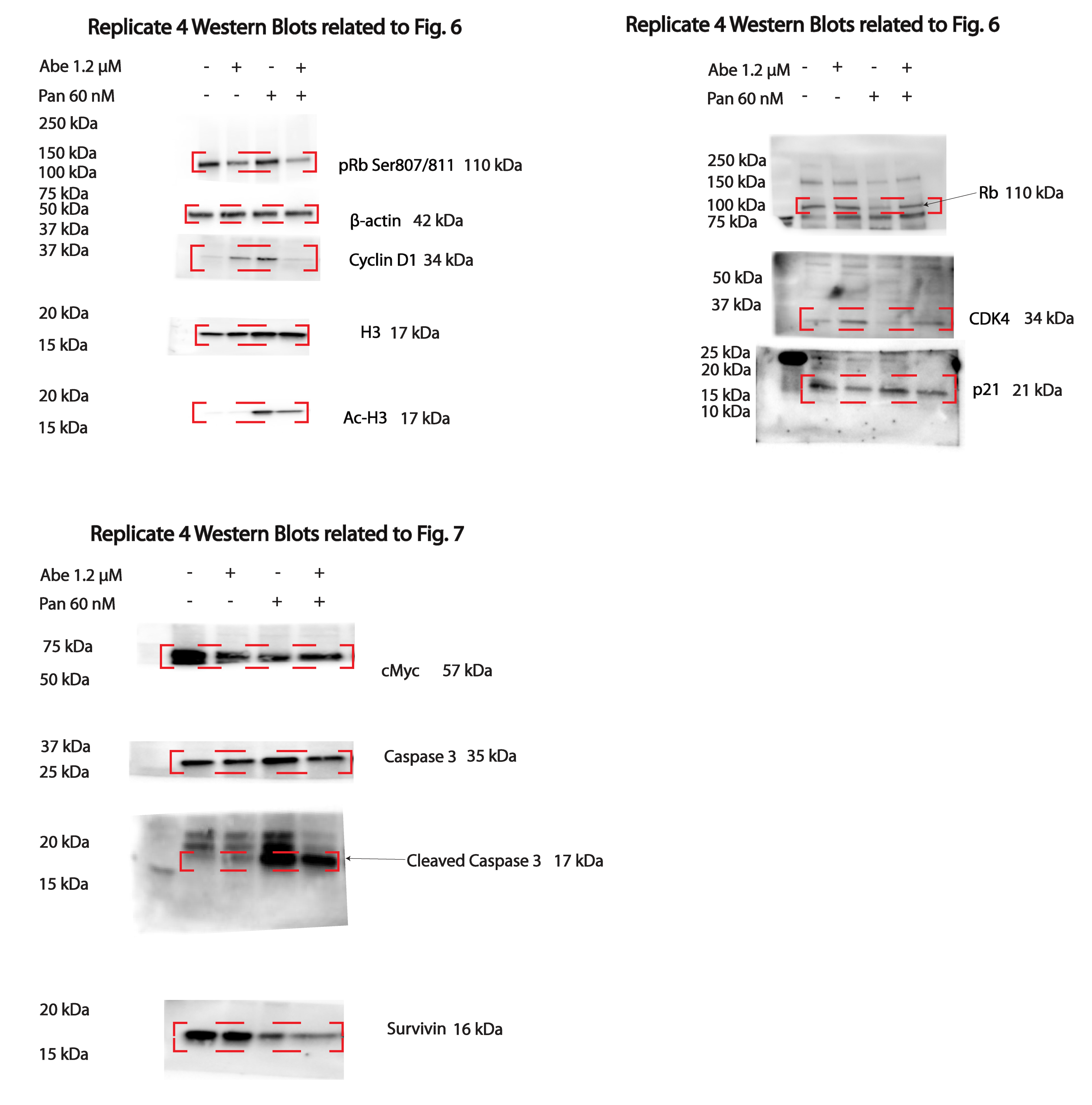

Supplement: Supplementary file 1 [file cancers-16-02713-s001.zip › Figure S5 Raw Western Blots Sup - Rep 4.tif]
